# Therapeutic effects of KRM-II-81, positive allosteric modulator for α2/3 subunit containing GABA_A_ receptors, in a mouse model of Dravet syndrome

**DOI:** 10.3389/fphar.2023.1273633

**Published:** 2023-10-02

**Authors:** Sachiko Nakakubo, Yasuyoshi Hiramatsu, Takeru Goto, Syuhei Kimura, Masashi Narugami, Midori Nakajima, Yuki Ueda, Hideaki Shiraishi, Atsushi Manabe, Dishary Sharmin, James M. Cook, Kiyoshi Egawa

**Affiliations:** ^1^ Department of Pediatrics, Hokkaido University Graduate School of Medicine, Kita-ku, Sapporo, Japan; ^2^ Department of Chemistry and Biochemistry, University of Wisconsin-Milwaukee, Milwaukee, WI, United States

**Keywords:** Dravet syndrome, mouse model, antiseizure, positive allosteric modulator for α2/3 subunit containing GABA A receptor, anxiolytics

## Abstract

**Introduction:** Dravet syndrome (DS) is an intractable epilepsy syndrome concomitant with neurodevelopmental disorder that begins in infancy. DS is dominantly caused by mutations in the *SCN1A* gene, which encodes the α subunit of a voltage-gated Na channel. Pre-synaptic inhibitory dysfunction is regarded as the pathophysiological mechanism, but an effective strategy for ameliorating seizures and behavioral problems is still under development. Here, we evaluated the effects of KRM-II-81, a newly developed positive allosteric modulator for α 2/3 subunit containing GABA_A_ receptors (α2/3-GABA_A_R) in a mice model of DS both *in vivo* and at the neuronal level.

**Methods:** We used knock-in mice carrying a heterozygous, clinically relevant *SCN1A* mutation (background strain: C57BL/6 J) as a model of the DS (*Scn1a* ^WT/A1783V^ mice), knock-in mouse strain carrying a heterozygous, clinically relevant *SCN1A* mutation (A1783V). Seizure threshold and locomotor activity was evaluated by using the hyperthermia-induced seizure paradigm and open filed test, respectively. Anxiety-like behavior was assessed by avoidance of the center region in locomotor activity. We estimated a sedative effect by the total distance traveled in locomotor activity and grip strength. Inhibitory post synaptic currents (IPSCs) were recorded from a hippocampal CA1 pyramidal neuron in an acutely prepared brain slice.

**Results:** KRM-II-81 significantly increased the seizure threshold of *Scn1a* ^WT/A1783V^ mice in a dose-dependent manner. A low dose of KRM-II-81 specifically improved anxiety-like behavior of *Scn1a* ^WT/A1783V^ mice. A sedative effect was induced by relatively high dose of KRM-II-81 in *Scn1a* ^WT/A1783V^ mice, the dose of which was not sedative for WT mice. KRM-II-81 potentiated IPSCs by increasing its decay time kinetics. This effect was more prominent in *Scn1a* ^WT/A1783V^ mice.

**Discussion:** Higher activation of α2/3-GABA_A_R by KRM-II-81 suggests a compensatory modification of post synaptic inhibitory function against presynaptic inhibitory dysfunction in *Scn1a* ^WT/A1783V^. The increased sensitivity for KRM-II-81 may be relevant to the distinct dose-dependent effect in each paradigm of *Scn1a* ^WT/A1783V^ mice.

**Conclusion:** Selective activation for α2/3-GABA_A_R by KRM-II-81 could be potential therapeutic strategy for treating seizures and behavioral problems in DS.

## 1 Introduction

Dravet syndrome (DS) is an early onset epileptic encephalopathy characterized by easily precipitated febrile seizure and a variety of refractory epileptic seizures including generalized convulsive seizures, focal seizures, myoclonic seizures, and atypical absence seizures (Gataullina and Dulac, 2017; Wirrell et al., 2022). The patients typically show normal development prior to the seizure onset, but manifest sever intellectual disability and symptoms of autism-spectrum disorders along with the development of refractory seizures (Sinoo et al., 2019).

A majority of the patients is caused by the heterozygosity of loss-of-function mutation in *SCN1A* gene encoding *a* subunit of the type 1 voltage-gated Na + channel (Nav1.1) (Claes et al., 2001). The phenotypes of DS including fever-induced seizure, epilepsy, intellectual disability and autistic behavior are well replicated by the mice carrying haploinsufficiency of *Scn1a* gene generated as an experimental model of DS (Ricobaraza et al., 2019). Prior works have shown that Nav1.1 is predominantly expressed in parvalbumin-positive, fast-spiking interneurons (PV + interneurons) (Ogiwara et al., 2007; Lorincz and Nusser, 2008) which make projection onto cell Soma or paroxysmal dendrites and constitute the robust perisomatic inhibition (Freund and Katona, 2007). In the mice model of DS, the decreased excitability of fast-spiking interneuron (Yu et al., 2006; Ogiwara et al., 2007) and reduced frequency of spontaneous inhibitory post-synaptic currents in pyramidal neurons (Han et al., 2012; Beretta et al., 2022)has been illustrated as a pathophysiology of DS. This insight is supported by clinical and experimental knowledge that epileptic features can be aggravated by anti-seizure drugs which blocks sodium channel blockers (Wirrell et al., 2022; Quinn et al., 2023). The precise mechanisms underlying the symptoms of DS might be more complex because recent studies provide the evidence that the electrophysiological properties of DS models can be altered depending on age or stage of symptomatic severity (Favero et al., 2018; Almog et al., 2021). Nevertheless, dysfunction of inhibitory systems should be one of the main cause of symptoms in DS.

Effective treatment for DS is still under development. The combination regimen including valproic acid, clobazam, styripentol, and topiramate is often described for preventing epileptic seizures (Cardenal-Muñoz et al., 2022; Wirrell et al., 2022). In addition, fenfluramine (Wijnen et al., 2023) and cannabidiol (Devinsky et al., 2017) are recently approved as adjunctive medications. Although these medications can ameliorate the severity of frequency of epileptic seizure, the remission of seizure is hardly obtained in the most case. Adverse effects including sedation are not negligible because a majority of patients receives multiple medication. Further, medications for improving intellectual disability or autistic behavior have not been established yet. Therefore, the development for novel compounds is still required for improving the quality of life in the patients with DS.

Because the impairment of fast spiking interneuron firing is regarded as one of the pathophysiological mechanisms of DS, an augmentation of GABA_A_ receptor-mediated inhibition would be the potential therapeutic strategies. Clobazam, one of the benzodiazepine class medications, is often used to treat epilepsy in patients with DS (Wirrell et al., 2022), and its efficacy has been shown in DS model mice (Hawkins et al., 2017; Quinn et al., 2023). GABA_A_ receptors are assembled from multiple subunit gene products and form mostly hetero-oligomeric pentamers (Olsen and Sieghart, 2008). Majority of synaptic GABA_A_ receptor subtypes are composed from two copies of a single α, two copies of a single β, and one copy of γ subunit. Accumulating evidence have revealed that each subunit combination has distinct distribution pattern and physiological function. *In vivo*, while α1 subunit-containing GABA_A_ receptors (α1-GABA_A_R) is responsible for the sedative and motor-impairment effects of positive allosteric modulator (PAM) for GABA_A_ receptors, the α2 and α3 subunit containing GABA_A_ receptors (α2/α3- GABA_A_R) mediate the anticonvulsant effects (Rivas et al., 2009; Rudolph and Knoflach, 2011) and anxiolytic effects (Löw et al., 2000, please also see Skolnick, 2012 for discussion). Distribution pattern of α2/3 subunit in the hippocampus or cortex is not fully uncovered. While previous studies reported that α2 subunit expression of CA1 pyramidal neuron was identical to receive projection from axo-axonic cells and parvalbumin-negative basket cells (Nyíri et al., 2001; Klausberger et al., 2002), such a cell type specific input has been challenged by subsequent studies showing its unbiased expression in the Soma (Kasugai et al., 2010; Kerti-Szigeti and Nusser, 2016). Further, electrophysiological evidence has indicated that activation of α2/α3- GABA_A_R is more prominent in perisomatic area rather than distal dendrites (Prenosil et al., 2006b; Nomura et al., 2019). Therefore, PAM for α2/α3- GABA_A_R might be suitable medication for DS patients compared to the non-selective PAM currently available for GABA_A_R, such as clobazam.

To date, several PAMs for α2/α3- GABA_A_R has been synthesized as anti-epileptic and/or anxiolytic compound (Atack, 2011; Chen et al., 2019), one of which has been shown to increase the threshold for heat-induced seizure in DS mice (Nomura et al., 2019). However, its therapeutic value for DS is still under evaluated because the efficacy for behavioral problem or functional property of α2/α3- GABA_A_R activation in DS model has not been uncovered yet. Imidizodiazepine, 5-(8-ethynyl-6-(pyridin-2-yl)-4H-benzo [f])imidazo[1,5-a][1,4]diazepin-3-yl)oxazole (KRM-II-81) is recently developed selective PAM for α2/α3- GABA_A_R (Poe et al., 2016) with sufficient oral bioavailability (Witkin et al., 2018). Its anti-epileptic effect has been shown in rodent models of epilepsy (Knutson et al., 2020). In this study, we investigated efficacy of KRM-II-81 on DS by using *Scn1a* deficit mice and illustrated that KRM-II-81 ameliorates hyperthermia-induced seizure susceptibility and anxiety-like behavior in these mice. Our findings provide an evidence that activation of α2/α3- GABA_A_R by KRM-II-81 is a potential therapeutic strategy for improving the symptoms of DS.

## 2 Materials and methods

### 2.1 Ethics approvals

All experimental procedures were carried out in accordance with the policies and protocols of Hokkaido University.

### 2.2 Animal

Mice were all kept in standard mouse cages, on a 12 h day and night cycle, and allowed free access to food and water, with temperature and humidity maintained at 23°C–25 °C and 45%–55%, respectively.

Mice carrying a heterogenous, clinically relevant *Scn1a* mutation on pure C57BL/6 J background (*Scn1a* ^WT/A1783V^ mice) were generated by crossing B6(Cg)-Scn1atm1.1Dsf/J mice (heterozygous for transgene, JAX strain #026133) and Cre-in a C57BL6/J background expressed Cre under the control of the ubiquitous CMV promoter from The Jackson Laboratory recombinase mice (B6.C-Tg (CMV-Cre)1Cgn/J; JAX strain #006054). These mice well represent phenotypes of DS including epilepsy, lower heating-induced seizure threshold and behavioral problems (Ricobaraza et al., 2019). P21-28 mice were used for hyperthermia-induced seizures palladium and slice electrophysiology experiments, while all behavioral experiments (open-filed test and grip strength) were performed at P60-90. Both male and female mice were included in all experiments.

### 2.3 Compounds

KRM-II-81 (5-(8-ethynyl-6-(pyridin-2-yl)-4H-benzo [f]imidazo[1,5-a][1,4]diazepin-3-yl)oxazole) was synthesized by G. Li, University of Wisconsin-Milwaukee, as described previously (Poe et al., 2016). For *in vivo* experiments, KRM-II-81 was suspended in 1% hydroxyethylcellulose/0.05% Tween 80/0.25% antifoaming agent in a volume of 1 mL/kg to administrate intraperitoneally (for open field test and grip strength measurement) or subcutaneously (for Hyperthermia-induced seizures paradigm). According to previous studies (Witkin et al., 2019). Application dosage was chosen either 0, 1.0, 5.0 or 10.0 mg/kg for all experiment. Selective activation for α2/3-GABA_A_R by application of the highest dose has been validated according to the preceding studies (Poe et al., 2016; Witkin et al., 2019). For electrophysiological recording, KRM-II-81 was suspended by dimethyl sulfoxide and applied via perfusion solution with final concentration 600 nM.

### 2.4 Hyperthermia-induced seizures

Hyperthermia-induced seizures paradigm was utilized to evaluate epileptogenic thermal threshold as previously described (Hawkins et al., 2017; Ricobaraza et al., 2019). Mice with postnatal days 21–28 (P21-28) received subcutaneous application of each drug 30 min before the hyperthermia protocol was initiated were acclimated to the rectal temperature probe (RET-3, World Precision Instruments) for 5 min. The mice were then placed into an acryl cylinder and heating was applied via inflated heating lamp (EXO TERRA heat glow infrared irradiation spot lamp, 150 W) from the top of the cylinder. The central body temperature was continuously monitored and heating power was adjusted to increase the temperature by 0.5 °C every 2 min. The recording was continued until the first clonic seizure with postural collapse occurred or until the temperature reached 45.0 °C.

### 2.5 Open field test (OF)

OF were performed as previously described in previous studies (Han et al., 2012; Egawa et al., 2023). Mice (P60-90) received an intraperitoneal administration of each drug 30 min before the initiation of experiment and were acclimated to the soundproofed testing room. Each mice was placed in the center of an empty arena (40 × 40 × 40 cm) and allowed to explore freely for 30 min. We analyzed the total distance traveled as well as the relative distance traveled in the center region (25 cm × 25 cm in the middle of the arena) in relation to the total distance traveled and time spent in the center region. The male/female ratio was enforced without significant difference.

### 2.6 Grip strength measurement

Forelimb grip strength of P60-90 mice was measured 30 min after administration of each drug intraperitoneally by using a grip strength meter (GPM-101B, Melquest).

### 2.7 Electrophysiology

Experiments were performed on acute hippocampal slices prepared from P21–28 *Scn1a* ^WT/A1783V^ mice or WT littermate mice in accordance with a previous works (Egawa et al., 2023). Mice were killed by decapitation under deep anesthesia using halothane or isoflurane, and brain coronal slices containing the hippocampus (350 μm thick) were cut on a microslicer (VF-300-0Z, Precisionary) in ice-cold modified artificial cerebrospinal fluid (ACSF) containing (in mM): 220 sucrose, 2.5 KCl, 1.25 NaH2PO4, 12.0 Mg2SO4, 0.5 CaCl2, 26.0 NaHCO3, and 30.0 glucose, pH 7.4 when gassed with 95% O2/5% CO2. The slices were kept in standard ACSF solution consisting of (in mM) 126 NaCl, 2.5 KCl, 1.25 NaH2PO4, 2.0 MgSO4, 2.0 CaCl2, 26.0 NaHCO3, and 20.0 glucose, pH 7.4 when gassed with 95% O2/5% CO2, at room temperature for over 1 h before experiments.

Slices were then transferred to a recording chamber on the stage of a microscope (Axioskop2, Zeiss) and continuously perfused with oxygenated ACSF at a flow rate of 2 mL/min at 30 °C. CA1 pyramidal neurons were visually identified on a monitor using a ×40 water immersion objective lens with an infrared differential interference contrast filter. The patch electrodes were pulled from borosilicate capillary tubing with a 1.5 mm diameter (GD-1.5; Narishige) with a horizontal puller P-97 (Sutter Instruments). The electrode resistance ranged from 4 to 6 MΩ. Signals were recorded using Axopatch 200 B (Molecular Devices), low-pass filtered at 2 kHz, and digitized at 6–10 kHz using a Digidata 1332A data acquisition system (Molecular Devices).

Whole-cell voltage-clamp recordings were performed under the presence of 6-cyano-7-nitroquinoxaline-2, 3-dione (CNQX; 20 µM), D-(−)-2-Amino-5-phosphonopentanoic acid (D-AP5; 50 µM), and CGP55845 (3 µM). CA1 pyramidal neurons were voltage-clamped at a holding potential of −60 mV using a pipette solution consisting of (in mM) 130 CsCl, 1 mM CaCl2, 2 MgCl2,10 HEPES-NaOH, 0.5 EGTA-KOH, 1.5 Mg-ATP, 0.5 mM Na2-GTP, and 2.5 QX314 (pH 7.3). Individual spontaneous inhibitory post synaptic currents (sIPSCs) were identified from 5-min current traces to analyze their frequency, peak amplitude, 10%–90% decay time using template search in pClamp 10 (Molecular Device). For the analysis of evoked inhibitory post synaptic currents (eIPSCs), a monopolar glass pipette (filled with ACSF) was placed in stratum pyramidale to evoke perisomatic synaptic responses and applied electric stimulation every 30 s with 200 µs in duration, and 100–400 pA in intensity. Three consecutive eIPSCs were averaged to analyze peak amplitude, 10%–90% decay time.

### 2.7 Data analysis

Log-rank tests with Bonferroni’s correction were used to compare cumulative seizure probability in hyperthermia-induced seizures paradigm. Threshold body temperature for hyperthermia-induced seizure in *Scn1a* ^WT/A1783V^ mice was compared among each KRM-II-81 dose by one-way analysis of variance (ANOVA) followed by Tukey’s post-hoc test. Two-way ANOVA followed by Šidák’s multiple comparison test was used to analyzing data obtained from OF test and grip strength test. Data from electrophysiological experiment were analyzed by two-way repeated measure ANOVA followed by Šidák’s multiple comparison test except comparison for cumulative probability (Kolmogorov-Smirnov test) and relative change of decay time between each genotype (unpaired *t*-test). Differences were determined to be significant when *p* < 0.05. Data are presented as mean ± standard error of mean. Statistical analysis was performed using Prism 9.0 (GraphPad software).

## 3 Results

### 3.1 KRM-II-81 raises the seizure threshold in *Scn1a* ^WT/A1783V^ mice

To estimate the anti-epileptic effect of KRM-II-81, we analyzed seizure susceptibility by using the hyperthermia-induced seizure assay. As shown previously, *Scn1a* ^WT/A1783V^ mice were prone to present hyperthermia-induced seizures with lower body temperature (38.4°C ± 0.21°C, n = 10) in comparison the littermate wild type mice (44.1°C ± 0.20°C, n = 7 out of 10 mice, *p* < 0.0001). While all of *Scn1a* ^WT/A1783V^ mice (n = 10) suffered seizures, 3 out of 10 wild type mice did not experience seizures when their body temperature reached to the maximum measurement. The seizure-induction probability is significantly higher in *Scn1a* ^WT/A1783V^ mice (*p* = 0.038 by χ2 test). These results validate the credibility of this assay for evaluating seizure-threshold in *Scn1a* ^WT/A1783V^ mice.

**FIGURE 1:**
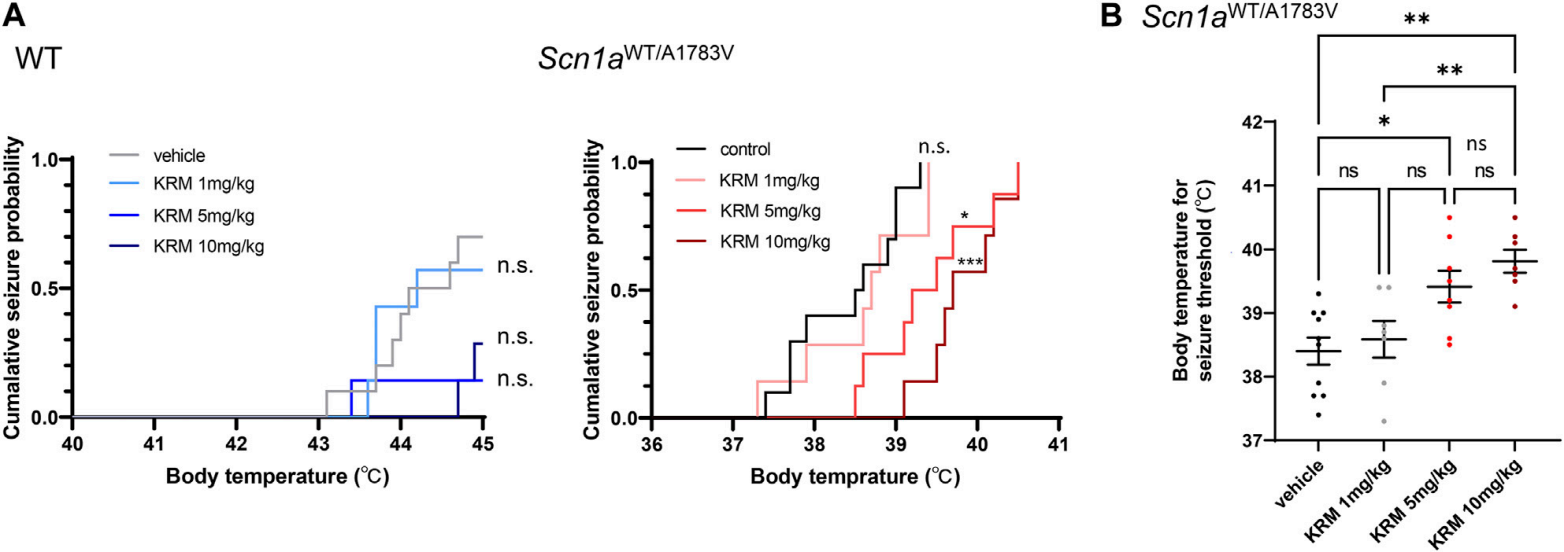
Effects of KRM-II-81 administration on hyperthermia-induced seizures **(A)**: Cumulative seizure probability against body temperature in WT (the left panel) and *Scn1a* ^WT/A1783V^ mice (the right panel). n. s.: Not significant, *: *p* < 0.05, ***: *p* < 0.001 compared to control by Log-rank test with Bonferroni correction **(B)**: Comparison of body temperature for seizure threshold by different doses of KRM-II-81 in *Scn1a* ^WT/A1783V^ mice). Ns: not significant, *: *p* < 0.05, **: *p* < 0.01 by Tukey’s post-hoc test. N = 7–10 for each group.

Systemic administration of KRM-II-81 significantly increased the seizure threshold of *Scn1a* ^WT/A1783V^ mice as shown by the right shift of the cumulative seizure probability curve for body temperature (*p* = 0.019 for 5mg/kg, *p* < 0.001 for 10mg/kg by log-rank test, Figure 1A). The seizure-inducing body temperature was significantly increased by 5mg/kg (*p* = 0.008) or 10mg/kg (*p* < 0.001) of KRM-II-81 (Figure 1B). There was no significant difference between 5 mg/kg and 10 mg/kg of KRM-II-81 (*p* = 0.456). In wild type mice, KRM-II-81 did not significantly alter the cumulative seizure probability curve at any doses (1mg/kg: *p* > 0.90, 5mg/kg: *p* = 0.140, 10mg/kg: *p* = 0.144). However, the total seizure-induction probability was decreased by 5mg/kg (14.2%, 1 out of 7 mice, *p* < 0.0001) or 10 mg/kg (28.6%, 2 out of 7 mice, *p* < 0.001 by χ2 test) of KRM-II-81 in comparison to vehicle control (70.0%, 7 out of 10 mice), while 1mg/kg not (71.4%, 2 out of 7 mice, *p* = 0.763). Indicating the anti-seizure effect of KRM-II-81 is not restricted to the pathogenic individuals in accordance with the preceding study.

### 3.2 Low dose KRM-II-81 rescues anxiety-like behavior of *Scn1a* ^WT/A1783V^ mice

**FIGURE 2:**
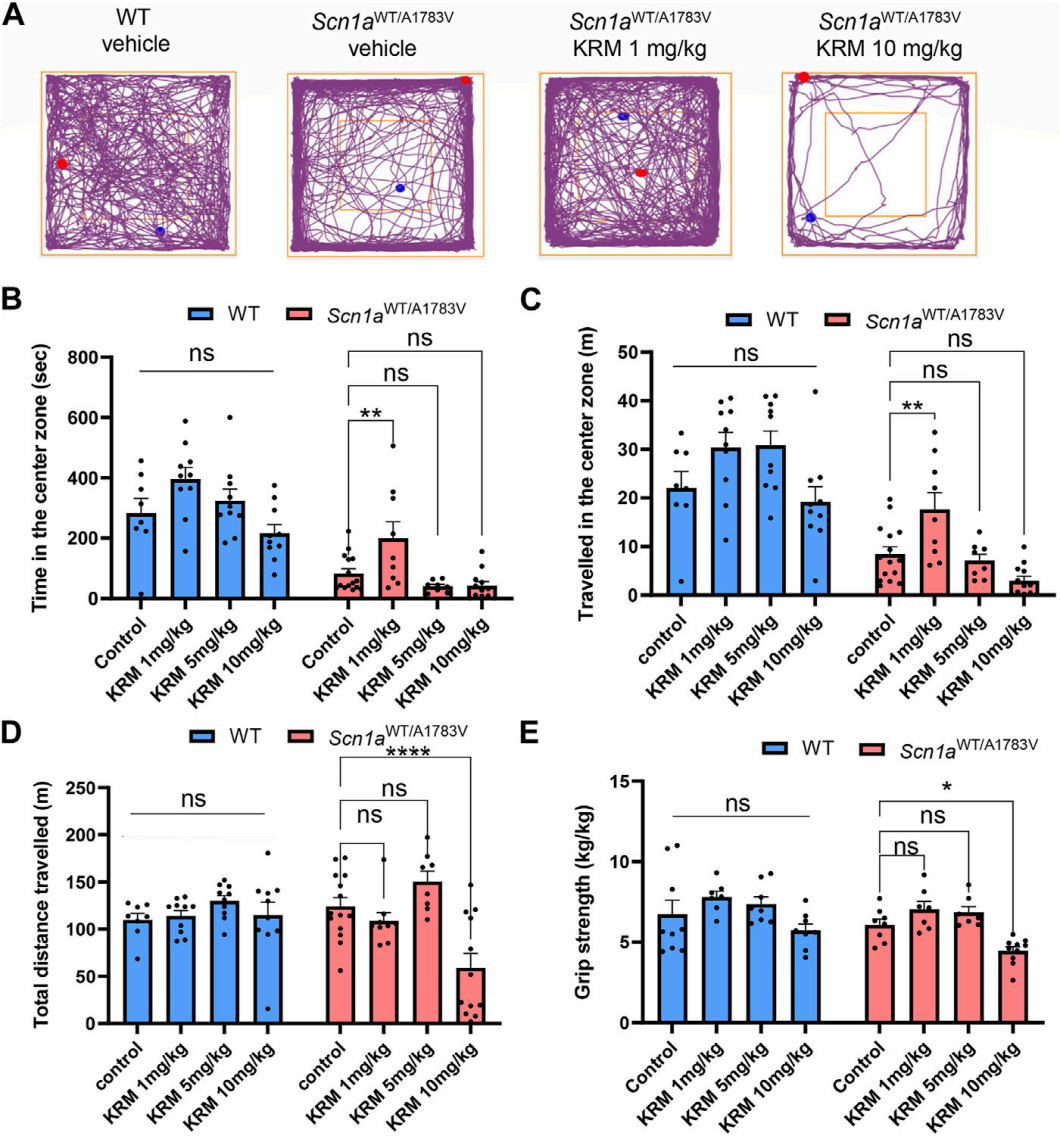
Effects of KRM-II-81 administration on the OF test and grip strength in *Scn1a* ^WT/A1783V^ mice **(A)**: Representative trajectory maps of the OF test in WT with vehicle, *Scn1a* ^WT/A1783V^ mice with vehicle, *Scn1a* ^WT/A1783V^ mice treated with 1 mg/kg of KRM-II-81 or with 10 mg/kg. The red and blue dots indicate the start and end points of the tracking, respectively **(B,D)**: Effects of KRM-II-81 on travelled length **(B)**, time **(C)** in the center zone and total distance traveled **(D)** in the OF test. N = 8–14 for each group **(E)**: Comparison of grip strength by different doses of KRM-II-81 in WT and *Scn1a* ^WT/A1783V^ mice, n = 7–10 for each group. Ns: not significant, *: *p* < 0.05, **: *p* < 0.01, ****: *p* < 0.0001 by Šidák’s multiple comparisons test after two-way ANOVA.

Previous studies have demonstrated that a selective agonist for α2 and/or α3 subunit-containing GABA_A_ receptors acts as anxiolytics without sedating effects (Atack et al., 2006). Thus, we next assess psychiatric effects of KRM-II-81 on *Scn1a* ^WT/A1783V^ mice by conducting OF test. KRM-II-81 was administered in doses of 1, 5 and 10 mg/kg at 30 min prior to the analysis as used in hyperthermia-induced seizure analysis. *Scn1a* ^WT/A1783V^ mice showed less time spent in the center region in compared to WT mice (main effect of factor genotype: F [1, 74] = 87.04, *p* < 0.001, Figures 2A,B). Distance traveled in the center region was similarly shorter in *Scn1a* ^WT/A1783V^ mice (main effect of factor genotype: F [1, 74] = 85.49, *p* < 0.0001, Figures 2A,C). These results were in accordance with the previous studies and represent an anxiety-like behavior of DS model mice (Han et al., 2012; Bahceci et al., 2020). KRM-II-81 had a significant main effect for both time and distance travelled in the center region (F [3, 74] = 9.688, *p* < 0.0001 and F [3, 74] = 9.655, *p* < 0.0001, respectively), while there were no significant interactions between dose and genotype (time: F [3, 74] = 1.065, *p* = 0.369, distance: F [3, 74] = 1.803, *p* = 0.154). *Post-hoc* analysis within each genotype revealed that 1 mg/kg of KRM-II-81 specifically increased time and distance travelled in the center region in *Scn1a* ^WT/A1783V^ mice (time in the center region, control vehicle v. s. 1mg/kg KRM-II-81: *p* = 0.007, control vehicle v. s. 5mg/kg KRM-II-81: *p* = 0.565, control vehicle v. s. 10 mg/kg KRM-II-81: *p* = 0.511, distance travelled in the center region, control vehicle v. s. 1mg/kg KRM-II-81: *p* = 0.003, control vehicle v. s. 5mg/kg KRM-II-81: *p* = 0.939, control vehicle v. s. 10 mg/kg KRM-II-81: *p* = 0.073, Figures 2A–C). In contrast, KRM-II-81 did not significantly affect these parameters in WT mice (time in the center region, control vehicle v. s. 1mg/kg KRM-II-81: *p* = 0.124, control vehicle v. s. 5mg/kg KRM-II-81: *p* = 0.791, control vehicle v. s. 10 mg/kg KRM-II-81: *p* = 0.480, distance travelled in the center region, control vehicle v. s. 1mg/kg KRM-II-81: *p* = 0.180, control vehicle v. s. 5mg/kg KRM-II-81: *p* = 0.152, control vehicle v. s. 10 mg/kg KRM-II-81: *p* = 0.853, Figures 2B,C). These results demonstrated that administration of low dose KRM-II-81 is specifically effective for improving anxiety-like behavior in *Scn1a* ^WT/A1783V^ mice.

### 3.3 Sedative dose of KRM-II-81 was lower in *Scn1a* ^WT/A1783V^ mice in compared to WT

KRM-II-81 showed significant main effect (F [3, 73] = 8.102, *p* < 0.0001) on total distance travelled in the open field test with significant interaction between dose and genotype (F [3, 73] = 5.358, *p* = 0.002), while there was no significant main effect of factor genotype (F [1,73] = 0.722, *p* = 0.398). In *Scn1a* ^WT/A1783V^ mice, either 1mg/kg or 5mg/kg of KRM-II-81 did not decrease the total distance (*p* = 0.611 and 0.215, respectively) but 10 mg/kg of KRM-II-81 did in comparison to control vehicle (*p* < 0.0001, Figures 2A,D). In contrast, any dose of KRM-II-81 did not reduce the total distance of WT mice (*p* = 0.987, 0.454, and 0.978 for 1mg/kg, 5mg/kg, and 10mg/kg in comparison to control vehicle, respectively, Figure 2D). The decreased locomotor activity by the higher dose of KRM-II-81 may be caused by its sedative effects in *Scn1a* ^WT/A1783V^ mice. To prove this possibility, we next evaluated the effect of KRM-II-81 administration on grip strength. A two-way ANOVA showed a significant main effect of KRM-II-81 on grip strength (F [3, 56] = 9.119, *p* < 0.0001), but there was not a significant interaction between factor genotype and factor drug (F [3, 56] = 0.286, *p* = 0.835). Similar to the result of total distance in the open field, 10 mg/kg of KRM-II-81 significantly decreased grip strength in *Scn1a* ^WT/A1783V^ mice (*p* = 0.042 in comparison to vehicle control), but not in WT mice (*p* = 0.3973, Figure 2E). The 1mg/kg or 5mg/kg dose of KRM-II-81 did not alter grip strength both in WT (*p* = 0.321 and 0.730 for 1mg/kg and 5mg/kg, respectively) and *Scn1a* ^WT/A1783V^ mice (*p* = 0.477 and 0.656, similarly, Figure 2E). These results indicate that the sedative dose of KRM-II-81 in *Scn1a* ^WT/A1783V^ mice is lower than that in WT.

### 3.4 KRM-II-81 more efficiently potentiated the inhibitory synaptic response in *Scn1a* ^WT/A1783V^ mice

Distinct dose-dependent effects of KRM-II-81 on the locomotor activity observed in *Scn1a* ^WT/A1783V^ mice implied that GABA_A_ receptor modification by KRM-II-81 might differ between WT and *Scn1a* ^WT/A1783V^ mice. To investigate this possibility, we analyzed inhibitory post synaptic currents (IPSCs) by utilizing whole-cell patch-clamp recordings from CA1 pyramidal neurons within acutely prepared brain slices. Due to the technical limitation of patch-clamp recording from full adult mice, we used juvenile mice (P21-28) in this experiment. GABA_A_ receptor-mediated spontaneous IPSCs (sIPSCs) were recorded under the blockage of AMPA, NMDA and GABA_B_ receptor receptors. Then, we applied 600 nM of KRM-II-81 in the perfusion solution. This concentration preserves the selectivity for α2/α3 subunit containing GABA_A_ receptors of KRM-II-81 (Lewter et al., 2017) and is assumed to be comparable to the brain plasma concentration obtained by administration of approximately 10mg/kg KRM-II-81 in rodents (Witkin et al., 2018).

**FIGURE 3:**
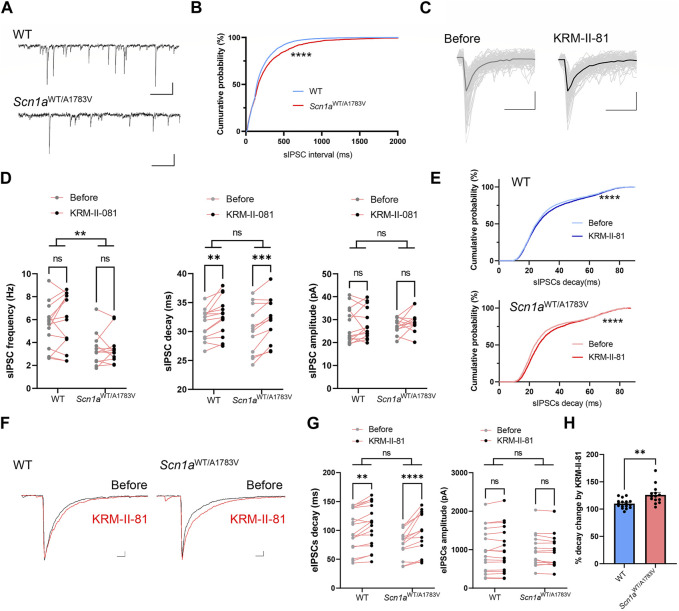
Effects of KRM-II-81 on inhibitory post synaptic currents (IPSCs) in the CA1 pyramidal neuron **(A)**: Representative trace of spontaneous inhibitory post synaptic currents (sIPSCs) recorded from a CA1 pyramidal neuron of WT (upper panel) and *Scn1a* ^WT/A1783V^ mice. Calibration:500 ms, 40 pA **(B)**: Cumulative probability curve for interval time of each sIPSCs analyzed from 14 neurons of WT and 11 neurons from *Scn1a* ^WT/A1783V^ mice. ****: *p* < 0.0001 by Kolmogorov-Smirnov test **(C)**: Overwrites of representative individual sIPSCs traces (thin gray lines) and their average traces (thick lines) obtained before and after application of KRM-II-81 into an acute slice from WT. Calibration:20 ms, 20 pA **(D)**: Effects of KRM-II-81 on frequency (left panel), decay (middle panel) and amplitude (right panel) of sIPSCs in WT and *Scn1a* ^WT/A1783V^ mice. *: *p* < 0.05, **: *p* < 0.01 by two-way ANOVA or subsequent post-hoc analysis. **(E)** Cumulative probability curve for decay time of each sIPSCs before and after an application of KRM-II-81 in WT (upper panel) and *Scn1a* ^WT/A1783V^ mice (lower panel). ****: *p* < 0.0001 by Kolmogorov-Smirnov test **(F)**: Representative evoked IPSCs (eIPSCs) traces before (black trace) and after (red trace) application of KRM-II-81 in WT (left panel) and *Scn1a* ^WT/A1783V^ mice (right panel). Calibration: 25 ms, 200 pA **(G)**: Effects of KRM-II-81 on frequency (left panel), decay (right panel) of eIPSCs in WT and *Scn1a* ^WT/A1783V^ mice. *: *p* < 0.05, ****: *p* < 0.0001 by post-hoc analysis after two-way ANOVA. n = 17 neurons for WT and 14 for *Scn1a* ^WT/A1783V^ mice. **(H)** Comparison of %decay time change by KRM-II-81 between WT and *Scn1a* ^WT/A1783V^ mice. **: *p* < 0.01 by unpaired *t*-test.

The frequency of sIPSCs was significantly decreased in *Scn1a* ^WT/A1783V^ mice in compared to WT as indicated by the right shift of the cumulative probability curve for interval time of sIPSCs (*p* < 0.0001 by Kolmogorov-Smirnov test, Figures 3A,B). Two-way repeated ANOVA for averaged value of sIPSCs frequency also indicated its decrement in *Scn1a* ^WT/A1783V^ mice (main effect of genotype: F [1, 24] = 9.739, *p* = 0.005, Figure 3D). Averaged decay time and peak amplitude of sIPSCs (Figure 3C) were comparable between WT and *Scn1a* ^WT/A1783V^ mice (main effect of genotype for decay: F [1, 24] = 0.482, *p* = 0.494, main effect of genotype for amplitude: F [1, 24] = 0.045, *p* = 0.884, Figure 3D). Similar decrement of sIPSCs in another *Scn1a* mutation mouse was previously shown (Yu et al., 2006; Han et al., 2012; Beretta et al., 2022) and could reflect the impairment of the presynaptic function of GABAergic neurons due to the loss of function of the *Scn1a* gene.

Administration of KRM-II-81 did not alter sIPSCs frequency or amplitude (main effect of drug for frequency: F [1, 18] = 0.638, *p* = 0.435, main effect of drug for amplitude: F F [1, 18] = 0.3028, *p* = 0.589), but significantly prolonged decay time kinetics in both WT and *Scn1a* ^WT/A1783V^ mice (main effect of drug: F [1, 18] = 20.22, *p* < 0.001, *post-hoc* analysis for WT: *p* = 0.009, for *Scn1a* ^WT/A1783V^ mice: *p* = 0.013, Figure 3D). Cumulative probability curve for decay time shifted toward the right by KRM-II-81 in both WT and *Scn1a* ^WT/A1783V^ mice (*p* < 0.0001 by Kolmogorov-Smirnov test for both genotype, Figure 3E). Prior work has illustrated that α2 subunit containing GABA_A_ receptor is dominantly distributed proximal to the somatic region (Prenosil et al., 2006a) and its activation potentiates IPSCs by increasing decay time kinetics, not by increasing amplitude (Nomura et al., 2019). Our results were in accordance with the preceding studies and indicates that KRM-II-81 surely acts as PAM for native GABA_A_ receptors. There was no significant interaction between factor genotype and factor drug for averaged decay time on each neuron (*p* = 0.699). Nevertheless, by referring to the cumulative probability curve (Figure 3E), KRM-II-81 preferentially affected sIPSCs with short to middle decay kinetics and its increment was more apparent in *Scn1a* ^WT/A1783V^ mice, which implies higher potentiation of GABA_A_ receptor by KRM-II-81 in these mice.

To assess the function of the α2/3-GABA_A_R more efficiently, we analyzed IPSCs evoked by perisomatic stimulation (eIPSCs) via a monopolar electrode placed on striatum pyramidale (Nomura et al., 2019). Similar to sIPSCs, eIPSCs decay time and amplitude was comparable between WT and *Scn1a* ^WT/A1783V^ mice (for decay, main effect of genotype: F [1, 23] = 2.482, *p* = 0.129, for amplitude, main effect of genotype: F [1, 23] = 0.097, *p* = 0.758). KRM-II-81 did not alter amplitude (main effect of drug: F [1, 23] = 0.737, *p* = 0.400, Figure 3G) but prolonged decay time in both WT and *Scn1a* ^WT/A1783V^ mice (main effect of drug: F [1, 23] = 42.64, *p* < 0.0001, post-hoc analysis for WT: *p* = 0.015, for *Scn1a* ^WT/A1783V^ mice: *p* = < 0.0001) with significant interaction between factor genotype and drug (F [1, 23] = 7.905, *p* = 0.010, Figures 3F,G). Relative change of decay time by KRM-II-81 was significantly higher in *Scn1a* ^WT/A1783V^ mice in comparison to WT mice (*p* = 0.007, unpaired *t*-test, Figure 3H). These results indicate that KRM-II-81 augments perisomatic inhibitory inputs onto CA1 pyramidal neurons more efficiently in *Scn1a* ^WT/A1783V^ mice than in WT.

## 4 Discussion

Seizures and behavioral problems in patients with DS are hardly relieved with existing medications although a majority of the patients takes their multiple combination regimen. Thus, it is essentially required to develop disease mechanism-based novel therapeutic strategy. In the present study, we focused on the strategy augmenting GABAergic synapse transmission because hypoexcitability of PV + interneuron has been proposed as a main pathophysiology in DS. PAM for α2/α3-GABA_A_R recently gathers an intensive attention as novel therapeutic promise to various medicinal target including epilepsy and anxiety. KRM-II-81 is the recently developed, selective PAM for α2/3-GABA_A_R (Poe et al., 2016), whose oral bioavailability is highly enough to reach sufficient brain concentration for occupying α2/3-GABA_A_R (Witkin et al., 2018). Its efficacy against epilepsies and psychiatric disorders including anxiety has been already predicted by preclinical studies using experimental models derived from wild type rodents (Poe et al., 2016; Witkin et al., 2018; Knutson et al., 2020). Therefore, we intended to evaluate the potential therapeutic value of KRM-II-81 in DS as well as the correlation between its efficacy and the disease pathophysiology.

We investigated therapeutic value of KRM-II-81 for epilepsy by utilizing hyperthermia-induced seizure assay because seizure precipitation by fever is one of the characteristic features of DS. *Scn1a* ^WT/A1783V^ mice had significantly lower seizure threshold in compared to WT as shown previously (Ricobaraza et al., 2019) and KRM-II-81 raised the threshold by dose dependent manner (Figure 1A). We could not analyze interaction between genotype and KRM-II-81 in the seizure-induction body temperature because a part of WT mice did not present seizure at a body temperature of the maximum measurement. Nevertheless, the results showing significant shift of the cumulative seizure probability curve in *Scn1a* ^WT/A1783V^ mice, but not in WT mice may suggest that anti-epileptic effect KRM-II-81 is more reliable in DS. Further studies using another seizure-induction paradigm may be required to clarify the presence of positive interaction between *SCN1A* gene mutation and the efficacy of KRM-II-81. Nevertheless, our results complement the previous study showing anti-convulsant effect of AZD7325 by single dose application in DS mice (Nomura et al., 2019) and highlight the therapeutic value of α2/3-GABA_A_R activation for reducing seizure susceptibility in DS.

Recent electrophysiological study have proposed that the pathophysiology of DS can be more complicated than “dysfunction of PV + interneuron” hypothesis because the decrement of its firing ability is observed transiently during developmental age in DS mice (Favero et al., 2018). According to their result, the age of mice we used in hyperthermia-induced seizure experiment (P21-28) corresponds to a transition stage from decreased to normal firing of PV + interneuron. Other previous study showed PV + interneuron firing is indeed impaired in this age (Ogiwara et al., 2007). Thus, further study would be required to clarify whether KRM-II-81 is similarly effective in adult age. *In vivo* imaging study have illustrated that dysfunction of PV + interneuron can still contribute to generating seizure in adult DS mice because its synchronous activity which precedes seizure was impaired in these mice (Tran et al., 2020). Because α2/3-GABA_A_R activation from fast spiking interneuron is important for network oscillation (Heistek et al., 2013), PAM for α2/α3-GABA_A_R could be promising medication in adult age of DS even if firing property of PV + interneuron is not impaired. This speculation should be validated in a future study investigating the reduction of spontaneous seizures through chronic application of KRM-II-81. Meanwhile, epileptic seizures in patients with DS are usually more prominent in childhood (Gataullina and Dulac, 2017). Therefore, the evidence for the anti-convulsant effect of KRM-II-81 provided in this study should retain a value for clinical implications, although the evidence is limited to juvenile mice.

In addition to refractory epilepsy, patients with DS are known to exhibit a variety of behavioral problems including anxiety as well as attention disorders (Sinoo et al., 2019). In DS model mice, the anxiety disorder is replicated by avoiding the center region in the locomotor activity (Han et al., 2012; Bahceci et al., 2020) (Figure 2A) and we have illustrated that this anxiety-like behavior is rescued by the low dose (1 mg/kg) application of KRM-II-81. Anxiolytic effects of KRM-II-81 has been previously shown in WT mice of the same strain we used in this study, but it was obtained by much higher dose (30 mg/kg) (Biggerstaff et al., 2020). In accordance with this, any dose of KRM-II-81 up to 10 mg/kg did not significantly alter the preference in the center region of WT mice in this study. Thus, our result indicates that dysregulation of inhibitory transmission via α2/3-GABA_A_R is involved in the mechanism underlying anxiety-like behavior in *Scn1a* deficit mice. Effects of KRM-II-81on anxiety-like behavior was obtained only when the low dose was administrated. The similar “inverted U″ dose effect was previously shown in other anxiolytics (Bruhwyler, 1990; Dielenberg et al., 1999; Smith et al., 2004), which might be partially explained by their sedative effects in higher dose. However, 5mg/kg of KRM-II-81 did not show anxiolytic effect although this dose did not induce any sedative effects. We did not provide any evidence to explain the reason, but the complexity of inhibitory signal processing network regarding regulation of anxiety (Babaev et al., 2018) might be responsible for the low dose-specific anxiolytic effect in our study.

Relatively high dose (10mg/kg) of KRM-II-81 induced sedative effects in *Scn1a* ^WT/A1783V^ mice. This result was unexpected because PAM for α2/3-GABA_A_R is usually characterized by its less sedative effect in compared to non-selective for GABA_A_R and specific activation for α2/3-GABA_A_R by our application protocol has been previously validated in rodents (Poe et al., 2016; Witkin et al., 2019). Indeed, previous study has shown that sedative effect of KRM-II-81 is lower than diazepam, it is not completely free though (Witkin et al., 2019). As its potential mechanism, we illustrated that positive modulation for α2/3-GABA_A_R by KRM-II-81 was amplified in *Scn1a* ^WT/A1783V^ mice in compared to WT, which could reflect compensatory plasticity mechanism (Luscher et al., 2011) resulting from pre-synaptic dysfunctions as shown by decreased sIPSC frequency (Figure 3B) (Yu et al., 2006; Han et al., 2012; Beretta et al., 2022) or by impairment of PV + neuron firing (Ogiwara et al., 2007). As a methodological limitation in this study, we evaluated cellular electrophysiology in juvenile mice, not fully adult mice we used for behavioral analyses, but the similar compensatory upregulation of GABA_A_ receptor expression under the disturbance of inhibitory activity has been often suggested in a variety of disorders in the central nervous system (Benes et al., 1992; Waldvogel and Faull, 2015; Pomares et al., 2020) in adulthood. Our results indicate that sensitivity for KRM-II-81 is increased in naïve individuals of *Scn1a* ^WT/A1783V^ mice and provide clinical implication that slow titration of PAM for α2/3-GABA_A_R would be particularly important for planning its administration protocol in future for DS. If the upregulation of α2/3-GABA_A_R function is compensatory mechanism, prone to the sedative effect might be resolved by chronic application of KRM-II-81. This insight should be investigated by future study utilizing chronic application protocol.

To date, a number of clinical trials utilizing PAM for α2/3-GABA_A_R has been conducted for treating anxiety disorder, but most of them are halted due to the lack of distinct therapeutic effects or unexpected adverse effects (Chen et al., 2019). KRM-II-81 is one of the recently developed PAMs for α2/3-GABA_A_R and now under preparation for future clinical trial to investigate efficacy of intractable epilepsy and anxiety disorders (Witkin et al., 2022). Our preclinical study indicates the potential therapeutic value of KRM-II-81 for improving epilepsy and anxiety-like behavior in DS. We did not evaluate its effects on other clinical problems, such as intellectual disability or sudden unexpected death; however, our findings may lead to novel, pragmatic therapeutic strategies for DS. For future clinical application, a pursuing for optimized dose should be important because efficient dose for anti-seizure effect and for anxiolytic effect was different in our acute application protocol. Further study investigating effects of chronic application of KRM-II-81 on a variety of phonotypes will be required to establish it.

## Data Availability

The original contributions presented in the study are included in the article/Supplementary Material, further inquiries can be directed to the corresponding author.
